# What We Know Currently about Mirror Neurons

**DOI:** 10.1016/j.cub.2013.10.051

**Published:** 2013-12-02

**Authors:** J.M. Kilner, R.N. Lemon

**Affiliations:** Sobell Department of Motor Neuroscience and Movement Disorders, UCL Institute of Neurology, London, UK, WC1N 3BG

## Abstract

Mirror neurons were discovered over twenty years ago in the ventral premotor region F5 of the macaque monkey. Since their discovery much has been written about these neurons, both in the scientific literature and in the popular press. They have been proposed to be the neuronal substrate underlying a vast array of different functions. Indeed so much has been written about mirror neurons that last year they were referred to, rightly or wrongly, as “The most hyped concept in neuroscience”. Here we try to cut through some of this hyperbole and review what is currently known (and not known) about mirror neurons.

## Main Text

### Introduction

Mirror neurons are a class of neuron that modulate their activity both when an individual executes a specific motor act and when they observe the same or similar act performed by another individual. They were first reported in the macaque monkey ventral premotor area F5 [1] and were named mirror neurons in a subsequent publication from the same group [2]. Ever since their discovery, there has been great interest in mirror neurons and much speculation about their possible functional role with a particular focus on their proposed role in social cognition. As Heyes [3] wrote “[mirror neurons] intrigue both specialists and non-specialists, celebrated as a ‘revolution’ in understanding social behaviour … and ‘the driving force’ behind ‘the great leap forward’ in human evolution…”. Indeed so much has been written in both peer-review literature and elsewhere about mirror neurons and their proposed functional role(s) that they have recently been given the moniker “The most hyped concept in neuroscience” [4].

For us, the discovery of mirror neurons was exciting because it has led to a new way of thinking about how we generate our own actions and how we monitor and interpret the actions of others. This discovery prompted the notion that, from a functional viewpoint, action execution and observation are closely-related processes, and indeed that our ability to interpret the actions of others requires the involvement of our own motor system.

The aim of this article is not to add to this literature on the putative functional role(s) of mirror neurons, but instead to provide a review of the studies that have directly recorded mirror neuron activity. To date, there have been over 800 published papers on mirror neurons (from a PubMed search using: “mirror neuron” OR “mirror neurons”). Here, we restrict our attention to only the primary literature on mirror neurons. Mirror neurons were originally defined as neurons which “discharged both during monkey’s active movements and when the monkey observed meaningful hand movements made by the experimenter” [2]. Thus, the key characteristics of mirror neurons are that their activity is modulated both by action execution and action observation, and that this activity shows a degree of action specificity. This distinguishes mirror neurons from other ‘motor’ or ‘sensory’ neurons whose discharge is associated with either execution or observation, but not both. It also distinguishes mirror neuron responses from other types of response to vision of objects or other non-action stimuli. As the activity of mirror neurons cannot yet be unambiguously detected using neuroimaging techniques, we have excluded human and non-human primate imaging studies from this review. We therefore focus on the 25 papers [1,2,5–27] that have reported quantitative results of recording mirror neurons or mirror-like neurons in macaque monkeys since 1992 (Table 1).

**Figure 1:**
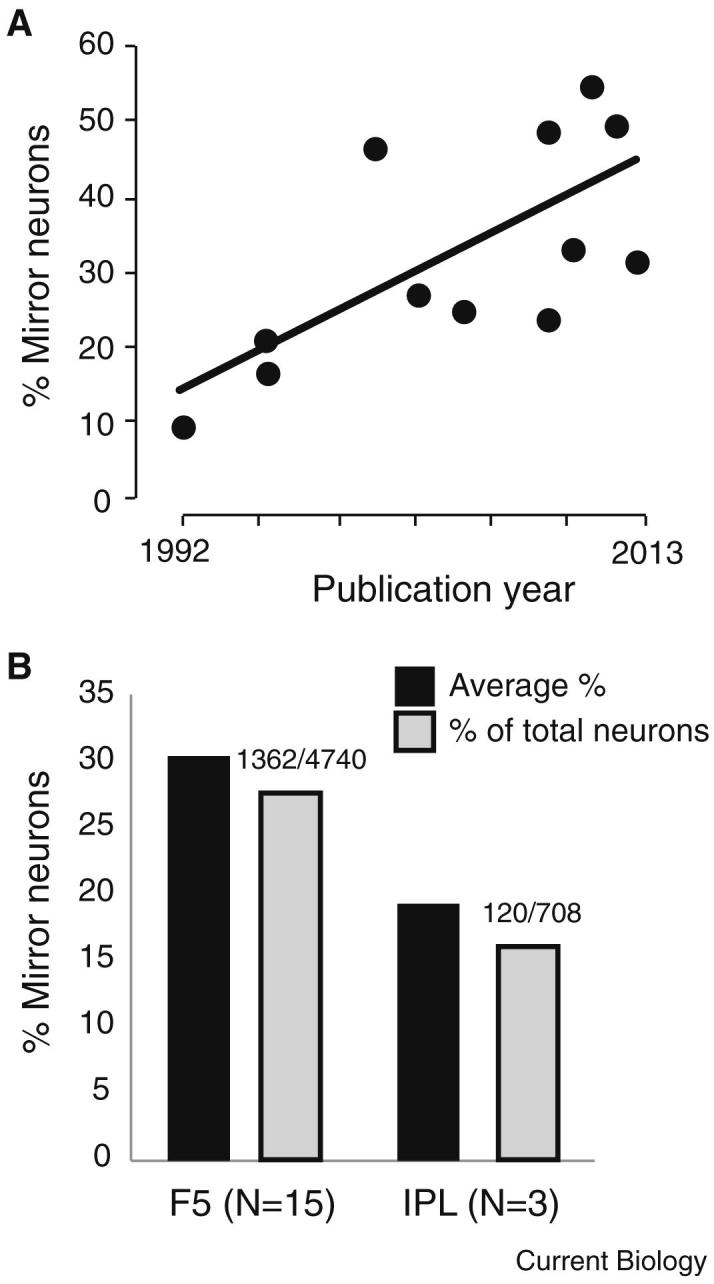
Number of mirror neurons recorded in areas F5 and in the IPL. (A) The percentage of mirror neurons as a function of publication year for studies reporting mirror neurons in F5 when observing hand actions. The black line shows the line of best fit. (B) The percentage of mirror neurons in premotor area F5 and in the inferior parietal lobule (IPL). The average percentage of mirror neurons for each region is shown in black and the percentage of total mirror neurons is shown in grey with the total number of mirror neurons and neurons recorded given above.

Mirror neurons were first described in the rostral division of the ventral premotor cortex (area F5) of the macaque brain, and have subsequently been reported in the inferior parietal lobule, including the lateral and ventral intraparietal areas, and in the dorsal premotor and primary motor cortex. But despite the large array of areas in which mirror neurons have been reported, the majority of mirror neuron research has studied the activity of mirror neurons in area F5 (15/25 papers; Figure 1A).

### Mirror Neurons in Ventral Premotor Region F5

Of the 15 papers reporting mirror neuron activity in area F5, 11 provide details of the number of mirror neurons recorded when observing the experimenter (not a video) reaching and grasping objects with their hand. On average, 33.6% of neurons recorded in F5 have been described as mirror neurons when the monkey observed hand actions performed by a human experimenter in front of them (ranging from 9.8–49.8%; Figure 1A,B). It is of note that the percentage of mirror neurons reported appears to increase as a function of time. This most likely reflects a sampling bias during data collection.

The first three papers [1,2,18] described the basic properties of mirror neurons, and their percentages are low compared with later studies. The more recent papers, in general, have investigated modulations of mirror neuron activity with some form of task manipulation. The methodological approach of these later papers is to first select neurons based on their motor properties (for example, selectivity for grasping) and then investigate the responses of this neuronal population to observed actions. This subtle change in the experimental strategy might explain the apparent increase in the percentage of mirror neurons in F5 as a function of time. Some investigators have avoided the sampling bias based on mirror properties by studying identified pyramidal tract neurons in area F5, selected on the basis of their antidromic response and not for their properties during action execution or observation [18]. A large proportion of pyramidal tract neurons in F5 and in M1 appear to show mirror-like responses (Table 1).

The three early papers [1,2,18] provided details about the relative selectivity of mirror neuron discharge during action execution and observation. On average, 48.9% of mirror neurons were classified as broadly congruent. Some mirror neurons discharged for only one action type, such as grasping, during both execution and observation, but showed no specificity for the type of grasp, for example precision grip or whole hand prehension. Others discharged for more than one type of observed action, for example grasping and holding. One of the three papers [2] describes a further category of mirror neurons, strictly congruent mirror neurons; these are defined as mirror neurons that respond selectively to one action type, such as precision grip, during both action execution and observation, and are reported as constituting 31.5% of mirror neurons recorded. Two of the three papers [2,18] report a further category of neuron in F5 that discharged during action observation but not during action execution; on average these neurons, which would not be included as mirror neurons, have been reported as making up 5.1% of the neurons in F5.

Further neuroanatomical studies of area F5 have revealed three interconnected sub-divisions [28]. The sub-division in which mirror neurons are located is suggested to be on the convexity of the precentral gyrus, adjacent to the inferior limb of the arcuate sulcus, and referred to as area F5c. This is distinguished from area F5p (posterior), which is reciprocally connected both with posterior parietal area AIP and primary motor cortex M1, and from area F5a (anterior) in the depth of the sulcus, which has prefrontal connections [29].

Two studies [7,9] have been reported that have shown that F5 mirror neurons discharged both to the observation of an action performed in front of the monkey by the experimenter and to videos of the same action. On average 26.9% of F5 neurons discharged when the monkey observed a video of a grasping action. One of the two studies [7] reported the relative number of mirror neurons that discharged to real and to videoed actions: 46.4% of neurons in F5 that responded to an executed action also responded when observing a real action, whereas only 22.3% responded when observing a videoed action. Although fewer mirror neurons responded when the monkey was observing the video of an action, for those mirror neurons that did discharge, there was no significant difference in the pattern or rate of mirror neuron discharge between real and videoed actions.

Two of the early papers [2,18] on mirror neurons reported that they could not find any neurons that discharged when monkeys observed an object being grasped with a tool. Subsequently, two studies [12,19] showed that mirror neurons did respond to such a tool-based action. In both these latter cases, however, the monkeys had received a high exposure to tool use during the training period prior to the recordings. One study [12] reported that 20% of F5 neurons were tool-responding mirror neurons, whereas the other reported the much higher percentage of 66.6% [20]. This high percentage most likely reflects a combination of a small sample size (n = 27) and strict inclusion criteria.

Two papers [15,16] have reported that neurons in F5 responded to the sound of an action: so-called auditory mirror neurons. On average, 17% of F5 neurons have been reported to have auditory properties (12.7% and 21.3%, respectively, in the two papers). Four papers [6–8,23] have reported that mirror neurons not only discharged during action observation but that their firing is further modulated by different factors: occlusion [23], relative distance of observed action [8], reward value [6] and the view point of the observed action [7]. Umilta *et al.* [23] showed that 19/37 mirror neurons discharged even when the observed action was occluded or hidden from the observer, demonstrating that direct vision of the action was not necessary to elicit mirror neuron discharge. Caggiano *et al.* [7] showed that 149/201 mirror neurons discharged preferentially for one or more of three different views of the same action (at 0, 90 and 180 degrees). Sixty of these neurons showed a preference for only one view point.

Caggiano *et al.* [8] also found that F5 mirror neurons have a preference for whether an observed action occurred in peripersonal or extrapersonal space: 27/105 mirror neurons discharged preferentially when the observed action occurred in the monkeys extra-personal space, whereas 28/105 mirror neurons discharged preferentially when the observed action occurred in the monkey's peri-personal space. The remaining 50 mirror neurons showed no preference. Caggiano *et al.* [6] reported that mirror neuron discharge is modulated by the value of the reward associated with the action: they showed that 40/87 mirror neurons responded more when a rewarded object was grasped, while 11/87 responded more when observing an action to a non-rewarded action. The remaining mirror neurons showed no preference.

One study [17] recorded from 64 neurons in F5 that were identified as pyramidal tract neurons. Thirty-one of these neurons were classified as mirror neurons, with 14/31 mirror neurons showing the ‘classic’ facilitation response during the action observation condition. Compared with baseline, the activity of the remaining 17 mirror neurons was significantly suppressed during action observation. The inclusion of these ‘suppression mirror neurons’ [8,17,24,25] clearly changes the overall proportion of neurons responsive during action observation.

In a recent study, Maranesi *et al.* [30] compared multiunit activity responses in areas F5, F4 (premotor regions) and F1 (primary motor cortex, M1). They reported a higher proportion of recording sites showing mirror type responses in area F5 (particularly in area F5c), compared with area F4 (caudal part of the ventral premotor cortex) and with F1. In addition, they reported that in penetration sites where they identified mirror responses, they were rarely able to evoke movement using intracortical microsimulation and argued that this might be due to presence of suppression mirror neurons, as first identified by Kraskov *et al.* [17].

One interesting study [27] looked at activity in premotor and parietal cortex neurons of the left hemisphere of a Japanese macaque monkey, either while it observed another monkey sitting opposite making reach-to-grasp movements for food rewards, or when it performed similar actions itself. Many neurons in both cortical areas were active during the other monkey’s movements, with the proportion varying across different actions (Table 1). Premotor cortex neurons showed a distinct preference for movements involving the observed monkey’s right arm and hand, and showed a similar preference for the monkey’s own right-sided actions.

### Mirror Neurons in the Inferior Parietal Lobule

Four papers [5,13,20,25] have reported neuronal activity recorded in the inferior parietal lobule that the authors have described as that of mirror neurons (Figure 1B). None of these papers explicitly specifies the percentage of neurons that were classified as mirror neurons; for three of these papers, however, we were able to estimate from the numbers in the papers that the average percentage of sampled neurons that were mirror neurons was 20% (41/165 Fogassi *et al.* [13]; 28/120 Bonnini *et al.* [5]; 51/423 Rozzi *et al.* [20]).

Two papers [5,13] describe the modulation of mirror neuron activity in the inferior parietal lobule by the overall goal of the observed action. Here monkeys observed an experimenter reaching for and grasping an object and either placing it in the mouth (eating) or placing it in a container (placing). On average 53% of mirror neurons had a significantly greater firing rate when the monkey observed the ‘eating’ compared with the ‘placing’ condition, 17% had a significantly greater firing rate for ‘placing’ compared with ‘eating’. The remaining 30% showed no difference between the two conditions. Yamazaki *et al.* [25] reported examples of mirror neuron activity in macaque area inferior parietal lobe; these neurons responded to the same action carried out in rather different contexts, suggesting that they are involved in encoding the ‘semantic equivalence’ of actions carried out by different agents in different contexts.

Rozzi *et al.* [20] investigated the properties of mirror neurons in the IPL. They reported that 58% of mirror neurons were responsive to only one type of hand action, for example grasping, and 25% were responsive to two different hand actions. The remaining 17% were responsive to either observed mouth actions or mouth and hand actions. Furthermore, they reported that 29% of IPL mirror neurons were strictly congruent and 54% were broadly congruent.

### Mirror Neurons in the Primary Motor Cortex

The first few papers [2,18] that described mirror neurons in area F5 also reported that the authors found no evidence of mirror activity in M1. Indeed, Gallese *et al.* [2] argued that, because most neurons in M1 show activity during self-movement, the absence of detectable mirror activity in M1 was evidence against the idea that this activity might actually represent monkey’s making small, covert movements while they watched the experimenter. Similarly, a recent multiunit recording study [29] found only a low level of mirror activity within primary motor cortex. However, three papers [10,22,24] have reported mirror neuron-like responses in M1.

Tkach *et al.* [22] reported that when monkeys either performed a visuomotor tracking task themselves, or watched the same target and cursor being operated by an experimenter, 70% (581/829) of recorded neurons in M1 showed stable preferred direction tuning during both execution and observation. These authors also reported that 60% (77/128) of neurons in dorsal premotor cortex were modulated in the same way.

Dushanova and Donoghue [10] recorded from neurons in M1 whilst the monkey either performed a point-to-point arm-reaching task or observed a human experimenter performing the same action. This study reported that 34.7% (105/303) of the neurons recorded in M1 were directionally tuned during both action execution and action observation. The mean firing rate during the observation condition was on average 46% of that during the execution condition. In addition, 38% of neurons retained the same directional tuning during both execution and observation conditions. It should be noted that these studies differ from those previously described that recorded from F5 and IPL.

All the studies on mirror neurons in F5 and IPL have employed tasks where the macaque monkey observed either a video or the experimenter performing simple reach and grasp actions. The two studies [10,22] described above on mirror-like responses in M1 differed in that they used tasks in which the monkey had been extensively trained on the motor execution task. It is unclear whether the relatively high percentage of these mirror-like responses, compared with those in F5 and IPL, reflects differences between the task or real differences in the number of mirror neurons.

The final paper [24] on M1 mirror neurons recorded from 132 neurons that were identified as pyramidal tract neurons; 58% of these neurons (77/132) were classified as mirror neurons. As in F5, these authors found that these pyramidal tract neurons were either facilitation mirror neurons (58.5%) or suppression mirror neurons (41.5%) during the action observation condition. In contrast to F5, facilitation mirror neurons in M1 fired at significantly lower rates during action observation vs execution, with the former reported as “less than half of that when the monkey performed the grip”. It is noteworthy that these authors made simultaneous EMG recordings from up to 11 different arm, hand and digit muscles and confirmed complete absence of activity during action observation.

### Mirror Neurons in Other Regions

Above, we have described the results of studies reporting mirror neurons in ventral premotor cortex, dorsal premotor cortex, primary motor cortex and inferior parietal lobule. Three further papers [14,21,26] have reported mirror neuron-like responses in two further areas. The first [14] recorded visuotactile bimodal neurons in the ventral intraparietal area (VIP). These are neurons that exhibit tactile receptive fields for a particular body part (for example, face or head) and also exhibit visual receptive fields in the congruent location. This study demonstrated that 48/541 bimodal neurons also exhibited visual receptive fields when observing the congruent area being touched on the experimenter. These neurons were not called mirror neurons but ‘body-matching bimodal neurons’.

Shepherd *et al.* [21] reported mirror neuron-like responses in the lateral intraparietal (LIP) area. These authors reported that 30/153 neurons in LIP responded not only when monkeys oriented attention towards the receptive field of those neurons, but also when they observed other monkeys orienting in the same direction.

Yoshida *et al.* [26] recently recorded from neurons in the medial frontal cortex, some of which selectively responded to self or observed actions within a social context. The neurons were recorded in one of two monkeys who, on alternate trials, chose a movement in order to earn a reward. Correct (or incorrect) choices rewarded (or punished: no reward) *both* monkeys. ‘Partner-type’ neurons were selectively responsive to the choices made by the other monkey, signalling the correct or incorrect choice made; interestingly around 19% of these ‘partner neurons’ showed decreased activity during self-movement.

### Relating Human Neuroimaging Data to Mirror Neuron Activity

Of the over 800 papers returned when searching PubMed for ‘mirror neurons’ or ‘mirror neuron’, the vast majority report the results of experiments on human subjects. Of these, the results of human neuroimaging experiments, specifically fMRI [31], confirm a broad overlap between cortical areas active in humans during action observation and areas where mirror neurons have been reported in macaque monkeys (see above). Thus, changes in the BOLD signal during action observation seem to be consistent with the existence of a mirror neuron system in humans, but they cannot yet furnish conclusive proof. There has, however, also been a report of single neuron activity recorded from human neurosurgical patients that has demonstrated mirror neuron activity [32]. Recordings were focused on medial frontal cortex and temporal lobe structures, and show the extensive nature of the mirror neuron system. Unfortunately, neither of the premotor or posterior parietal areas so heavily investigated in monkeys were available for study in these patients.

Central to being able to interpret human fMRI studies of the mirror neuron system is understanding the relationship between the BOLD signal in human and mirror neuron activity in macaque monkey. To this end, monkey fMRI studies have now demonstrated significant activity during action observation in regions where mirror neurons have been previously reported [33,34]. These monkey imaging studies have taken advantage of enhancing the neurovascular responses with an iron-based (MION) contrast agent. As with the vast majority of human fMRI studies, however, there is difficulty in relating these results to mirror neurons, in that they only employ an action observation condition and have no action execution condition. This makes it difficult to calibrate the activity changes in observation to those in execution, and also raises the possibility that sensory responses other than mirror responses contribute to the neurovascular changes (see Introduction).

One possible way of attributing the fMRI response to a single neuronal population, such as mirror neurons, is to use fMRI adaptation, or repetition suppression. This is a neuroimaging tool that has been adopted to identify neural populations that encode a particular stimulus feature [35]. The logic behind fMRI adaptation is that neurons decrease their firing rate with repeated presentations of the stimulus feature that those neurons encode. By extension it has been argued that the BOLD signal will also decrease with repeated presentations. It has been argued that areas of the cortex that contain mirror neurons should show fMRI adaptation both when an action is executed and subsequently observed, and when an action is observed and subsequently executed. This is because the stimulus feature encoded in mirror neurons is repeated irrespective of whether the action is observed or executed [36].

The results of such studies have produced mixed results. Of the five studies using this technique published to date [36–40], only three have demonstrated significant fMRI adaptation consistent with the presence of mirror neurons in the human brain [38–40]. One possible explanation for the mixed results is that humans do have mirror neurons, but that they do not alter their pattern of activation when stimuli that evoke their response are repeated. Indeed a recent study [9] has shown some evidence that mirror neurons may not alter their firing rate during repetitions of the same action; however, in this work the neuronal activity represented in the local field potential (LFP) did modulate with repetition. Further work is clearly required to determine why the BOLD signal in humans and the LFP in monkeys do adapt with repetition, while the evidence to date suggests that mirror neurons may not.

Great care must be taken when comparing the results from human and monkey studies. Specifically, readers must pay careful attention to the difference in the level of inference between the different modalities. The majority of human neuroimaging studies report significant results at the population level where the variance is estimated across subjects. This is in contrast to the studies reporting mirror neurons in macaque monkeys, where the aim is to test whether individual neurons show a consistent modulation of firing rate during periods of action observation and execution. Here the inference is closer to the analysis of fMRI at the single subject level. Therefore, when it is reported that 30% of neurons in any region were significantly modulated during both action observation and execution this does not mean that the remaining 70% do not modulate at all. Rather, it means there was not sufficient statistical evidence that these neurons displayed mirror activity. Indeed it is quite possible that when tested at the population level, the neurons that are non-significant at the single neuron level could be significantly modulated when observing an action.

The point here is that care must be taken when arguing that ‘only’ X% of neurons in any brain region are mirror neurons. The ‘only’ implies that the remaining neurons are not significantly modulated in any way during action observation. This is not a valid inference as to do so would be to accept the null hypothesis. This may be particularly problematic for cortical regions where responses in individual mirror neurons are relatively weak, such as in M1.

It is often assumed that mirror neuron activity during action observation is driven, bottom-up, by the visual (or auditory) input. The review of mirror neuron discharge presented here provides evidence that this is, at best, an incomplete description of mirror neuron firing. We now know that mirror neuron firing rates are modulated by view point [7], value [6] and that they even discharge in the absence of any visual input [23]. This suggests that mirror neurons can be driven or modulated top-down by backward connections from other neuronal populations. Indeed, the requirement for such top-down input to regions containing mirror neurons was realized by Jacob and Jeannerod [41], who argued that it was impossible for a mirror neuron system driven uniquely by the visual input to correctly infer an intention from an observed action if two or more different intentions would generate the same action. The fact that mirror neurons can be driven by backward connections is consistent with recent predictive coding models of mirror neuron function [42–44]. Within this framework, mirror neurons discharge during action observation not because they are driven by the visual input but because they are part of a generative model that is predicting the sensory input. This framework provides a theoretical account of mirror neuron activity that resolves the one-to-many mapping problem described by Jacob and Jeannerod [41] and is consistent with top-down modulation of mirror neuron firing rates.

### Concluding Remarks

The discovery of mirror neurons has had a profound effect on the field of social cognition. Here we have reviewed what is currently known about mirror neurons in the different cortical areas in which they have been described. There is now evidence that mirror neurons are present throughout the motor system, including ventral and dorsal premotor cortices and primary motor cortex, as well as being present in different regions of the parietal cortex. The functional role(s) of mirror neurons and whether mirror neurons arise as a result of a functional adaptation and/or of associative learning during development are important questions that still remain to be solved. In answering these questions we will need to know more about the connectivity of mirror neurons and their comparative biology across different species.

## Figures and Tables

**Table 1:** Proportion of neurons recorded in macaque premotor cortex (area F5) and posterior parietal cortex that showed mirror neuron properties.

Reference	Recording area	No. neurons	No. mirror	% mirror	1Action specificity	Observed effector
Bonini *et al.*[5]	F5	154	36	23.4%	y	Hand
Caggiano *et al.*[6]	F5	299	149	49.8%	n	Hand
Caggiano *et al.*[8]	F5	219	105	48%	n	Hand
Caggiano *et al.*[7]	F5	224	123	54.9%	n	Hand (video)
Caggiano *et al.*[9]	F5	785	247	31.5%	n	Hand (video)
Ferrari *et al.*[11]	F5	485	130	26.8%	y	Mouth
Ferrari *et al.*[12]	F5	209	52	24.9%	y	Hand
Gallese *et al.*[2]	F5	532	92	17.3%	y	Hand
Kohler *et al.*[16]^2^	F5	497	63	12.7%	y	Auditory
Kraskov *et al.*[17]	F5	64	31	48.4%	y	Hand (PTNs)
di Pellegrino *et al.*[1]	F5	184	18	9.8%	y	Hand
Rizzolatti *et al.*[18]	F5	300	60	20%	y	Hand
Rochat *et al.*[19]	F5	282	92	32.6%	y	Hand
Umilta *et al.*[23]	F5	220	103	46.8%	y	Hand
Bonini *et al.*[5]	IPL	120	28	23.3%	y	Hand
Fogassi *et al.*[13]	IPL	165	41	24.8%	y	Hand
Rozzi *et al.*[20]	IPL	423	51	12%	y	Hand
Shepherd *et al.*[21]	LIP	153	30	19.6%	n	Eye-gaze
Dushanova and Donoghue [10]	M1	303	105	34.6%	y	Reaching
Tkach *et al.*[22]	M1	829	581	70.1%	y	Tracking arm
Vigneswaran *et al.*[24]	M1	132	77	58.3%	n	Hand (PTNs)
Tkach *et al.*[22]	PMd	128	77	60.1%	y	Tracking arm
Ishida *et al.*[14]	VIP	541	48	8.9%	y	Bimodal tactile/visual
Fujii *et al.*[27]	PM^3^	148	_	3–14%^4^	n	Hand
	IPS^5^	148	-	10–42%^4^	n	

1 This column indicates if mirror neurons were tested for any form of action specificity.

2 These data were further analysed by Keysers *et al*. [15].

3 Including area F5.

4 See text.

5 Included anterior bank of the intraparietal sulcus (IPS). PTN, pyramidal tract neuron.
